# Investigating the use of cuprizone and lysolecithin to model demyelination *ex vivo* in sagittal rat brain organotypic slice cultures

**DOI:** 10.3389/fncel.2025.1609806

**Published:** 2025-05-22

**Authors:** Brooke Hawker, Bronwen Connor, Amy McCaughey-Chapman

**Affiliations:** Department of Pharmacology and Clinical Pharmacology, Centre for Brain Research, School of Medical Sciences, Faculty of Medical and Health Sciences, University of Auckland, Auckland, New Zealand

**Keywords:** demyelination, remyelination, corpus callosum, sagittal brain slice culture, lysolecithin, cuprizone

## Abstract

**Introduction:**

The development of organotypic slice cultures of central nervous system (CNS) tissues has bridged the gap between simple in vitro cell cultures and complex in vivo whole animal studies. Organotypic brain slice cultures are a useful tool to study neurological disease, providing a more complex 3-dimensional system than standard 2-dimensional in vitro cell culture. In particular, organotypic brain slice cultures provide an excellent model to study the processes of demyelination and remyelination associated with neurological disease and injury. However, organotypic brain slice cultures are typically generated using coronal sectioning or regionspecific hippocampal or cerebellar tissue. We have previously reported the ability to generate sagittal organotypic brain slice cultures, allowing us to investigate the anterior-to-posterior integrity of the corpus callosum during demyelination and remyelination processes. To extend our sagittal organotypic brain slice culture model, this study compares the ability for two common demyelinating agents, cuprizone (CPZ) or lysolecithin (LPC), to induce demyelination of the corpus callosum.

**Methods:**

Rat brain sagittal organotypic slice cultures were generated with clear visualization of the corpus callosum and treated either with CPZ (1 mM) or LPC (0.5 mg/mL).

**Results:**

We demonstrate that CPZ treatment induces acute demyelination followed by endogenous remyelination 1-week post-treatment. Conversely, we show that LPC treatment results in prolonged demyelination of the corpus callosum that is maintained 5 weeks post-treatment and is associated with an acute astroglia response.

**Discussion:**

Overall, this study demonstrates the use of CPZ and LPC to model either acute or prolonged demyelination in a sagittal organotypic brain slice culture system. These models provide a platform for studying acute and chronic demyelination and for testing new therapeutic approaches aimed at enhancing remyelination prior to conducting in vivo experiments.

## Introduction

Inflammatory demyelinating diseases (IDDs) are neurological disorders characterized by damage to the myelin sheath, the protective layer surrounding neuronal axons in the central nervous system (CNS), the most common of which is multiple sclerosis (MS). The myelin sheath acts as an insulating shield, facilitating the rapid propagation of action potentials as well as providing structural and trophic support to neurons (Bradl and Lassmann, 2010). Demyelination occurs when the myelin sheath is damaged, an action that disrupts nerve signal conduction and increases neuronal vulnerability to degradation. Remyelination is a reparative process in which resident oligodendrocyte precursor cells (OPCs) differentiate into mature oligodendrocytes (Ols), which then wrap around and myelinate axons, restoring the myelin sheath. However, remyelination is often inefficient in pathological environments, resulting in incomplete remyelination and persistent neurological deficits (Chari, 2007).

Developing effective therapies to target and enhance remyelination requires robust model systems that can effectively replicate the pathological processes occurring and provide platforms for therapeutic testing. The predominant pre-clinical models of demyelination are *in vivo* animal models such as the experimental autoimmune encephalomyelitis (EAE) or cuprizone (CPZ) mouse model (Torre-Fuentes et al., 2020). However, the development of *ex vivo* slice culture models has provided an alternative model that bridges the gap between *in vitro* and *in vivo* models, enabling the investigation of demyelination and remyelination under controlled conditions, with the added advantage of reduced cost and ethical considerations (Humpel, 2015).

Organotypic slice cultures are slices of whole tissue cultured to preserve 3-dimensional cytoarchitecture and anatomical organization, retaining cellular interactions between neighboring cells and proteins of the extracellular matrix (Humpel, 2015). Introduced in the 1980s, organotypic slice cultures were traditionally cultured using the roller-tube method but have since been optimized to culture via the air-liquid interface method, which allows slices to simultaneously receive nutrients from the culture medium and humidified air, facilitating long-term viability (Gähwiler, 1981; Stoppini et al., 1991). Organotypic slice cultures of CNS tissues are well established within the literature, with successful models reported representing the whole brain, cortex, hippocampus, cerebellum and spinal cord (Ullrich et al., 2011; Kaplanis et al., 2023; Staal et al., 2011; Götz and Bolz, 1992; Skrede and Westgaard, 1971; Bonnici and Kapfhammer, 2008). In this case, organotypic brain slice cultures are considered advantageous as they allow experimental manipulation of intact CNS regions, such as demyelination of the corpus callosum through treatment with various compounds.

CPZ (*bis*-cyclohexanoneoxalyhydrazone) is a well-established demyelinating toxicant, inducing consistent and widespread demyelination *in vivo* following 6 weeks of daily 0.2–0.3% CPZ treatment that can be reversed by endogenous remyelination occurring on cessation of treatment (Blakemore, 1972; Hiremath et al., 1998; Skripuletz et al., 2011). CPZ toxicity is linked to the induction of oxidative stress pathways, mitochondrial dysfunction and subsequent apoptosis of mature oligodendrocytes, causing demyelination *in vivo and in vitro*. However, the role of CPZ in *ex vivo* systems remains unexplored (Pasquini et al., 2007; Bénardais et al., 2013; Martínez-Pinilla et al., 2021). Establishing the effect of CPZ on organotypic brain slice culture could provide a crucial platform for evaluating therapeutic interventions prior to *in vivo* studies. Lysolecithin (LPC) is another well-characterized demyelinating agent, commonly used both *in vivo* and *ex vivo* (Torre-Fuentes et al., 2020). LPC exerts toxicity through disruption to lipids within the myelin sheath, increasing membrane permeability, compromising myelin integrity, leading to eventual breakdown and loss of oligodendrocytes (Plemel et al., 2018). As such, LPC is considered directly toxic to mature, myelin-producing oligodendrocytes as opposed to non-myelinating cells of the oligodendroglial lineage. However, their differentiation is suggested to be disrupted by LPC (El Waly et al., 2014). The use of LPC to model demyelination *ex vivo* was first demonstrated in cerebellar slices, and we recently demonstrated the ability of LPC to induce demyelination in *ex vivo* longitudinal spinal cord slices (Birgbauer et al., 2004; Hawker et al., 2024).

Traditionally, the predominant regions modelled using organotypic brain slice cultures are the cerebellum, hippocampus, and occasionally coronal whole-brain sections (Humpel, 2015). Extending this technology, we have previously demonstrated the application of sagittal organotypic brain slice cultures to model 6-hydroxydopamine (6-OHDA), a-amino-3-hydroxy-5-methyl-4-isoxazolepropionic acid (AMPA) and quinolinic acid-mediated cell death (McCaughey-Chapman and Connor, 2017; McCaughey-Chapman and Connor, 2022). Generating slices in the sagittal plane provides clear visualization of the heavily myelinated corpus callosum and hence provides a key region of interest in studying the processes of demyelination and remyelination. Additionally, the corpus callosum is a well-documented region of demyelination in CPZ and LPC *in vivo* models and is affected in MS patients (Steelman et al., 2012; Yazdi et al., 2015; Simon et al., 1986).

The current study builds on our established sagittal organotypic brain slice culture platform to model demyelination of the corpus callosum using CPZ or LPC. We show that CPZ treatment induces acute demyelination followed by endogenous remyelination 1 week post-treatment. Conversely, we show that LPC-treatment results in prolonged demyelination of the corpus callosum that is maintained 5 weeks post-treatment and is associated with an acute astroglia response. Overall, this study demonstrates the use of CPZ and LPC to model acute or prolonged demyelination, respectively, in sagittal organotypic whole brain slice cultures. These models provide a valuable platform for testing new therapeutic approaches aimed at enhancing remyelination prior to conducting *in vivo* experiments.

## Materials and methods

### Animals

Postnatal 9- to 11-day old male Sprague–Dawley rat pups (*n* = 7) were used in this study. All animals were housed in a 12-h light–dark cycle with access to food and water ad libitum. Animal euthanasia was in accordance with the New Zealand Animal Welfare Act 1999 and was approved by the University of Auckland Animal Ethics Committee (approval #R22387). All efforts were made to minimize the number of animals used. The work has been reported in line with the ARRIVE guidelines 2.0.

### Rat sagittal brain organotypic slice culture generation and culturing

Sagittal brain organotypic slice cultures were generated and cultured as previously described (McCaughey-Chapman and Connor, 2017; McCaughey-Chapman and Connor, 2022). Preliminary studies were conducted to determine the optimal orientation of slice cultures for the experiment. Sagittal sectioning was preferred based on the larger area of the corpus callosum in slices, allowing for easier identification and analysis (Supplementary Figure S1). Briefly, animals were euthanized by decapitation. To extract the brain, skin was removed from the back of the head and the skull was peeled back exposing the brain which was carefully removed and cut into two hemispheres. A single hemisphere was mounted onto a vibratome chuck [Lecia Biosystems] with superglue. The brain was sectioned in the sagittal plane at 300 μm thickness until the corpus callosum was no longer visible, generating 5–6 slices per hemisphere. During sectioning, the vibratome chamber was filled with ice-cold medium consisting of Advanced Dulbecco’s Modified Eagle Medium/Ham’s F-12 (DMEM/F-12) with 1% penicillin–streptomycin [Thermo Fisher Scientific, #12634010 and #15140148]. Individual slices were mounted onto sterile membrane inserts in 6-well plates [Corning, #COR3450] and cultured at the air-membrane interface at 35°C with 5% CO_2_. Slices were cultured in Minimum Essential Medium (MEM) with Hanks balanced salts [Thermo Fisher Scientific, #11575032], 1% penicillin–streptomycin and 25% horse serum [Thermo Fisher Scientific, #16050130] for 3 days, with 1 mL of medium added below the membrane insert. To limit glial scar formation, a cocktail of three mitotic inhibitors was added to the medium for the first 3 days of culturing: uridine, 5-fluorodeoxyuridine and cytosine-ß-arabinofuranoside [4.4 mM each, Sigma, #U3003, #F0503 and #C1768]. The slices were then transitioned into a serum-free medium consisting of Advanced DMEM/F-12 with 2% B-27 supplement [Thermo Fisher Scientific, #17504044] and 1% N-2 supplement [Thermo Fisher Scientific, #17502048] and cultured for up to 6 weeks. All slices were cultured for 7 days prior to treatment to allow for complete myelination of the tissue (Birgbauer et al., 2004).

### Slice culture treatment

Cuprizone powder (*bis*-cyclohexanoneoxalyhydrazone) [Sigma, #C9012] was dissolved in 50% ethanol to a concentration of 0.1 M at 60°C for 20 min with shaking, until complete dissolution. To obtain the final working concentration (1 mM, established after preliminary studies), the stock solution was diluted in culture medium for each treatment. Cuprizone stock solution was prepared fresh for each treatment. The concentration of Cuprizone used was established following preliminary studies, which were based on previous *in vitro* studies (Martínez-Pinilla et al., 2021; Pasquini et al., 2007). TNFa [Peprotech, #300-01A] and IFNg [Peprotech, #300–02] were used at a final concentration of 50 ng/mL, based on Pasquini et al., 2007. Lipopolysaccharide [Thermo Fisher Scientific, #17502048] was used at a concentration of 15 mg/mL based on Di Penta et al. (2013). Rapamycin [Tocris, #1292] was dissolved in dimethyl sulfoxide (DMSO) [Sigma, #D4540], generating a stock solution of 1 mM. To obtain the final working concentration of 15 ng/mL (Tyler et al., 2009) stock solution was diluted in culture medium.

To assess the effect of CPZ on demyelination, slices were treated for 72 h with either 1 mM CPZ, 1 mM CPZ + cytokines (15 ng/mL Tumour Necrosis Factor-a (TNFa) + 15 ng/mL Interferon-g (IFNg)), 1 mM CPZ + 15 mg/mL LPS, cytokines alone or 15 mg/mL LPS alone. To assess the effect of chronic CPZ exposure on demyelination, slices were treated every 2 days for 1 or 2 weeks. In the CPZ + rapamycin experiment, slices were treated every 2 days for 1 week. Following treatment removal, all slices were maintained in serum-free medium for an additional 24 h, 1 week or 2 weeks prior to fixation and immunohistochemical analysis.

Lysolecithin (LPC) [Sigma, #62962] was dissolved in 100% sterile methanol to a stock concentration of 50 ng/mL. To obtain the final working concentration of 0.5 mg/mL, the stock solution was diluted in culture medium. To investigate the effect of LPC, slices were incubated for 17 h in 0.5 mg/mL LPC, based on the study by Birgbauer et al. (2004). Thereafter, slices were cultured for an additional 24 h, 72 h, 1 week, 3 weeks or 5 weeks, in serum-free culture medium until fixation for immunohistochemical analysis, as previously described (Hawker et al., 2024).

### Immunohistochemistry

Slices were fixed in 4% paraformaldehyde for 24 h at 4°C (Haydar et al., 1999; McCaughey-Chapman and Connor, 2017). Whole mount slices were stained using a shortened version of the optical clearing technique iDISCO (Renier et al., 2014), as previously described (McCaughey-Chapman and Connor, 2022; Hawker et al., 2024). Briefly, after 24 h of permeabilization, the slices were blocked and then incubated in primary antibody for 48 h. The extent of demyelination and remyelination was assessed through quantification of myelin protein expression. Slices were co-labelled with Myelin Basic Protein (MBP) [1:100, MAB386] and Myelin Oligodendrocyte Glycoprotein (MOG) [1:250, ab233549] (*n* = 3 slices per timepoint and treatment). The presence of astrocytes in cultures was assessed through quantification of the expression of Glial Fibrillary Acidic Protein (GFAP) [1,250, G3893]. Alexa Fluor conjugated secondary antibodies were used to fluorescently label the antigens of interest and following dehydration in a series of increasing concentrations of methanol and cleared using dibenzyl ether, the membrane-bound slices were placed onto a glass microscope slide and imaged using a Zeiss LSM-800 inverted confocal microscope.

### Quantification and statistical analysis

The extent of MBP, MOG and GFAP staining was assessed through measuring the integrated density in ImageJ, following consistent background subtraction and threshold adjustment across all images. Three images were captured from the genu and body of the corpus callosum for each slice, captured at 607 mm × 607 mm. The total MBP+, MOG + and GFAP+ density was calculated for each slice. An average per timepoint and treatment group was calculated (*n* = 3 slices). The MBP+, MOG + and GFAP+ fluorescence data was reported as a percentage of the fluorescence intensity in untreated slices. Statistical analysis was performed using SPSS Statistics (IBM, USA). Statistical significance was determined using raw values, with either a one-way ANOVA or a two-way ANOVA followed by post-hoc analysis using Bonferroni corrections in the case of a significant ANOVA.

## Results

### CPZ-induced demyelination in *ex vivo* rat sagittal brain slice cultures is not enhanced with exogenous activation of inflammation

**Figure 1:**
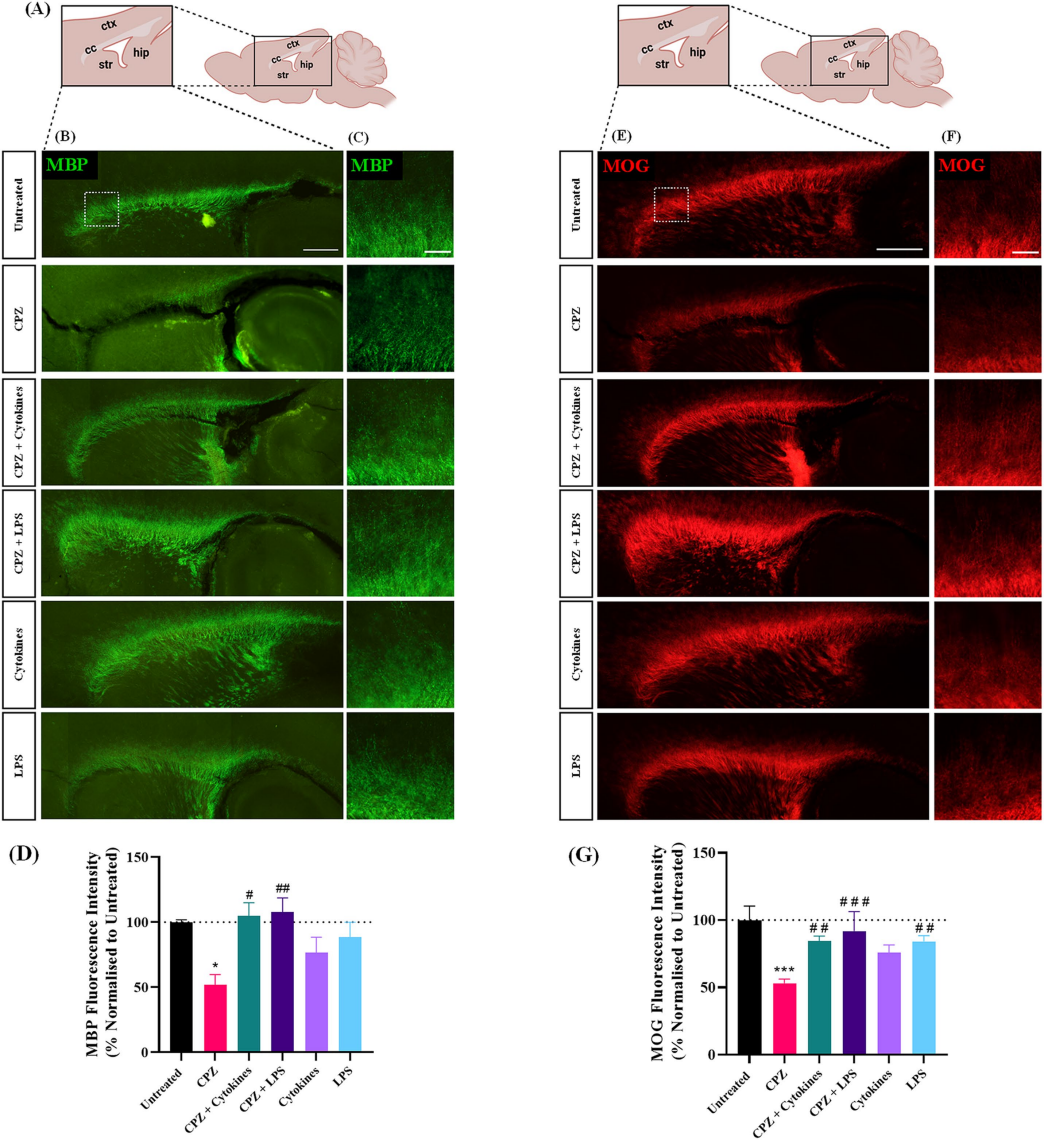
Treatment of sagittal brain slice cultures with 1 mM cuprizone induces demyelination. **(A)** Schematic diagram of sagittal brain slice indicating the area of interest, namely the corpus callosum. **(B,C)** MBP and **(E,F)** MOG staining in the corpus callosum of sagittal brain slice cultures following 72 h treatments with 1 mM cuprizone (CPZ), 1 mM CPZ + Cytokines (15 ng/mL TNFa + 15 ng/mL IFNg), 1 mM CPZ + 15 mg/mL LPS, cytokines or LPS. **(B,E)** Scale bar: 750 μm. **(C,F)** Scale bar: 150 μm. **(D)** Quantification of the mean MBP fluorescence intensity in untreated or treated slices. Data are presented as a percentage of MBP expression in untreated slices 24 h post-treatment removal. Data represent mean ± SEM with *n* = 3. Statistical significance was determined by a one-way ANOVA with Bonferroni’s *post-hoc* test with * for *p* < 0.05 indicating a significant effect of CPZ treatment compared to untreated slices. A significant effect of treatment compared to CPZ-treated slices was depicted by # for *p* < 0.05 and ## for *p* < 0.01. **(G)** Quantification of the mean MOG fluorescence intensity in untreated or treated slices. Data are presented as a percentage of MOG expression in untreated slices 24 h post-treatment removal. Data represent mean ± SEM with *n* = 3. Statistical significance was determined by a one-way ANOVA with Bonferroni’s *post-hoc* test with *** for *p* < 0.001 indicating a significant effect of CPZ treatment compared to untreated slices. A significant effect of treatment compared to CPZ-treated slices was depicted by ## for *p* < 0.01 and ### for *p* < 0.001. ctx: cortex, cc: corpus callosum, str: striatum, hip: hippocampus.

To assess the effect of a 72-h treatment of CPZ with or without the exogenous addition of inflammatory stimulators, the expression of myelin proteins MBP and MOG was examined and quantified 24 h post-treatment in slices either untreated or treated with CPZ, CPZ + cytokines (TNFa + IFNg), CPZ + LPS, cytokines alone or LPS alone (Figure 1A). MBP and MOG staining was seen in all treatment groups along the myelinated corpus callosum (Figures 1B,C,E,F). Quantification of MBP + fluorescence intensity revealed a significant effect of treatment on MBP expression (*p* = 0.0045). Subsequent *post hoc* analysis demonstrated a significant reduction in mean MBP expression in slices treated with CPZ in comparison to untreated slices (Untreated: 100% ± 1.44%, CPZ-treated: 51.89% ± 6.45%, *p* = 0.038). Furthermore, compared to CPZ treatment, there was a significant increase in MBP expression with CPZ + cytokine treatment (104.8% ± 8.27%, *p* = 0.015) and CPZ + LPS treatment (107.67% ± 9.0%, *p* = 0.008) (Figure 1D). Quantification of MOG + fluorescence intensity also revealed a significant effect of treatment on MOG expression (*p* = 0.00004). Subsequent post hoc analysis demonstrated a significant reduction in mean MOG expression in slices treated with CPZ in comparison to untreated slices (Untreated: 100% ± 8.5%, CPZ-treated: 53.08% ± 2.52%, *p* = 0.00002). Furthermore, compared to CPZ treatment, there was a significant increase in MOG expression with CPZ + cytokine treatment (84.6% ± 2.97%, p = 0.008), CPZ + LPS treatment (91.85% ± 11.75%, *p* = 0.0005) and LPS treatment alone (84.19% ± 3.46%, *p* = 0.009) (Figure 1G).

Taken together, these results indicate that the treatment of sagittal brain slice cultures with 1 mM CPZ for 72 h induces demyelination within the corpus callosum, an effect that is not seen either when CPZ is co-administered with exogenous inflammatory stimulators, or in response to exogenous inflammatory stimulators alone.

### Cuprizone treatment induces acute demyelination of the corpus callosum

**Figure 2:**
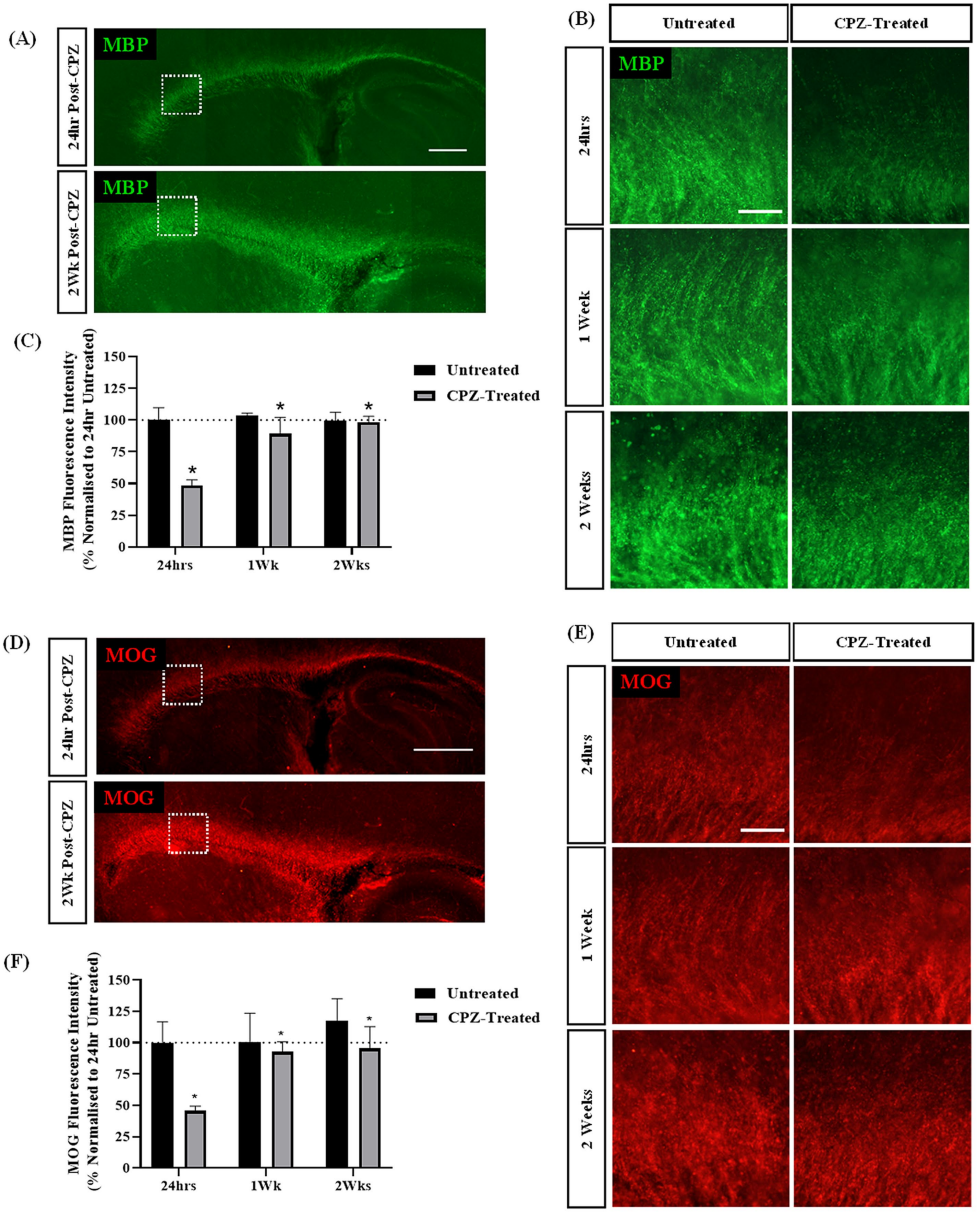
Cuprizone-induced demyelination in sagittal brain slice cultures is restored 2-weeks post-treatment. **(A,B)** MBP and **(D,E)** MOG staining in the corpus callosum of sagittal brain slice cultures following treatment with 1 mM CPZ for 72 h. **(A,D)** Scale bar: 750 mm. **(B,E)** Scale bar: 150 mm. **(C)** Quantification of the mean MBP fluorescence intensity at 24 h, 1 and 2 weeks following treatment with 1 mM CPZ. Data are presented as a percentage of MBP expression in untreated slices 24 h post-treatment. Data represent mean ± SEM with *n* = 3. Statistical significance was determined by a two-way ANOVA with a simple main effect of treatment with * for *p* < 0.05. **(F)** Quantification of the mean MOG fluorescence intensity at 24 h, 1 week and 2 weeks following treatment with 1 mM CPZ. Data are presented as a percentage of MOG expression in untreated slices 24 h post-treatment. Data represent mean ± SEM with *n* = 3. Statistical significance was determined by a two-way ANOVA with a simple main effect of treatment with * for *p* < 0.05.

To further assess the effect of CPZ-treatment on sagittal brain slice cultures over a longer period of time, slices were treated with CPZ for 72 h and the expression of myelin proteins MBP and MOG were quantified at 24 h, 1 week and 2 weeks post-treatment. MBP and MOG staining was identified in all slices along the corpus callosum (Figures 2A,D). Higher magnification images suggested a reduction in MBP and MOG expression in the corpus callosum 24 h post CPZ-treatment (Figures 2B,E). Quantification of MBP + fluorescence intensity revealed a significant effect of treatment on MBP expression, irrespective of time (*p* = 0.014) (Figure 2C). CPZ-treated slices had a mean MBP fluorescence intensity of 48.40% ± 3.74% at 24 h, 89.35% ± 10.5% at 1-week post-treatment and of 98.29% ± 3.76% at 2 weeks post-treatment compared to untreated slices at 24 h. A similar effect on MOG + fluorescence intensity was found with a significant effect of treatment on MOG expression, irrespective of time (*p* = 0.016) (Figure 2F). CPZ-treated slices had a mean MOG fluorescence intensity of 45.68% ± 3.07% at 24 h, 92.8% ± 6.4% at 1-week post-treatment and of 95.49% ± 14.11% at 2 weeks post-treatment compared to untreated slices at 24 h.

Together, these results indicate that the treatment of sagittal brain slice cultures with 1 mM CPZ for 72 h induces acute demyelination as measured by a reduction in both MBP + and MOG + fluorescence intensity at 24 h. However, after 1 and 2 weeks in culture, MBP and MOG levels appear to be restored.

### Chronic CPZ treatment does not induce demyelination

**Figure 3:**
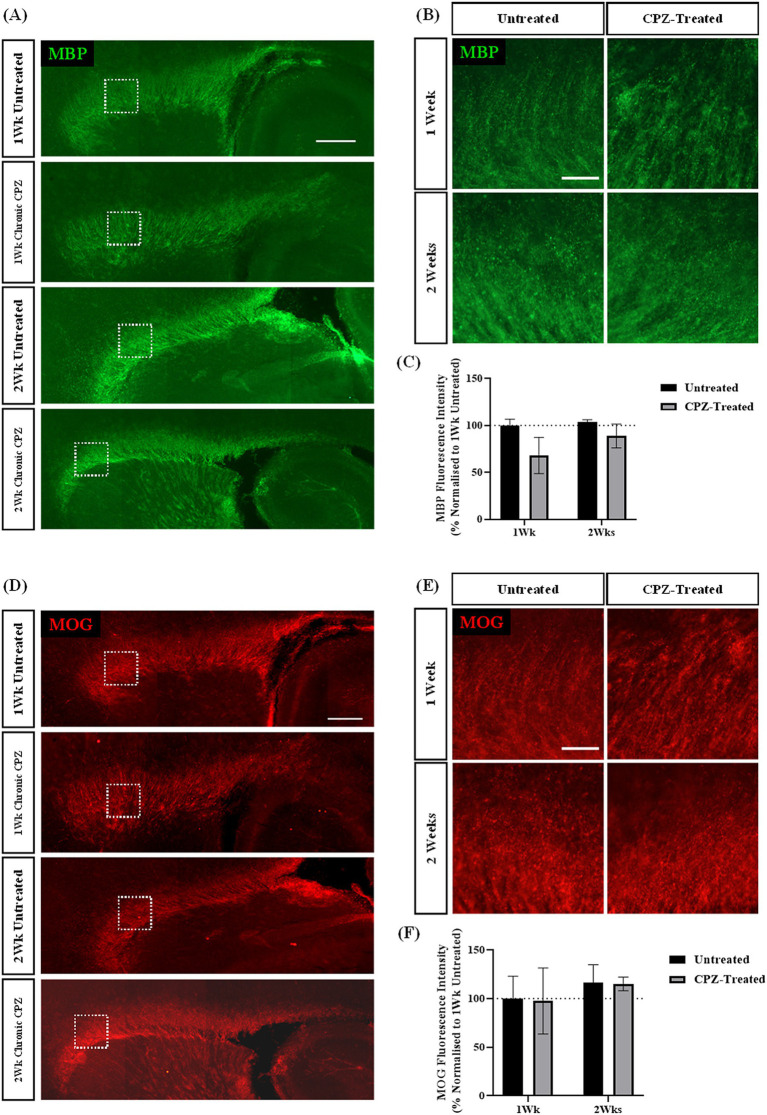
Chronic cuprizone treatment does not result in demyelination. **(A,B)** MBP and **(D,E)** MOG staining in the corpus callosum of sagittal rat brain slice cultures following chronic treatment with 1 mM CPZ for 1 or 2 weeks. **(A,D)** White box indicates region of quantification. Scale bar: 750 mm. **(B,E)** Scale bar: 150 mm. **(C)** Quantification of the mean MBP fluorescence intensity after 1 or 2 weeks of chronic 1 mM CPZ treatment. Data are presented as a percentage of MBP expression in untreated slices at 1 week. Data represent mean ± SEM with *n* = 3. No statistical significance was determined by a two-way ANOVA. **(F)** Quantification of the mean MOG fluorescence intensity after 1 week or 2 weeks of chronic 1 mM CPZ treatment. Data are presented as a percentage of MOG expression in untreated slices at 1 week. Data represent mean ± SEM with *n* = 3. No statistical significance was determined by a two-way ANOVA.

To assess if a longer treatment paradigm would provide significant maintenance of demyelination, we investigated whether treatment of slices with CPZ for either 1- or 2- weeks induced chronic reduction in the expression of MBP and MOG. The expression of myelin proteins MBP and MOG were assessed at 1- and 2- weeks post-treatment. We identified MBP and MOG staining in the corpus callosum of all treatment slices (Figures 3A,B,D,E). Quantification of MBP + fluorescence intensity revealed 67.95% ± 15.7% MBP fluorescence intensity following 1 week of CPZ treatment and 88.79% ± 10.38% MBP fluorescence intensity following 2 weeks of CPZ treatment. However, there was no significant effect of treatment or time in culture on the MBP fluorescence intensity (*p* = 0.497) (Figure 3C). Quantification of MOG + fluorescence intensity showed a mean MOG fluorescence of 97.53% ± 27.81% following 1 week of CPZ treatment and 115.141% ± 5.78% following 2 weeks of CPZ treatment. Again, there was no significant effect of treatment or time in culture on the MOG fluorescence intensity (*p* = 0.982) (Figure 3F).

These results indicate that treatment of slices for either 1- or 2- weeks with 1 mM CPZ was not effective at inducing demyelination compared to the extent of demyelination seen after 72 h exposure to 1 mM CPZ.

### Co-treatment of cuprizone with rapamycin does not prevent acute remyelination seen with cuprizone alone

**Figure 4:**
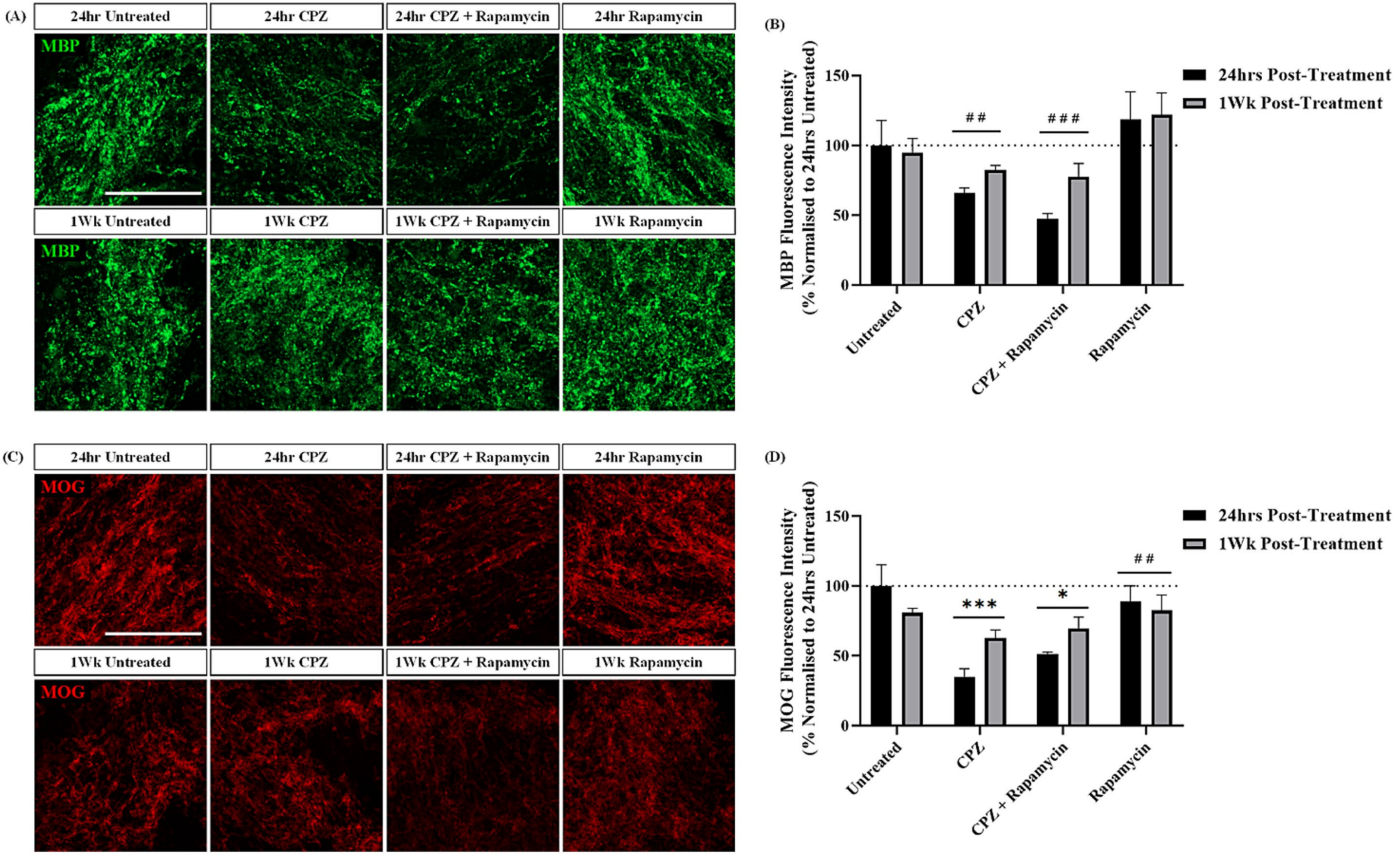
Rapamycin treatment does not prevent remyelination following cuprizone treatment. **(A)** MBP and **(C)** MOG staining in the corpus callosum of sagittal rat brain slice cultures following treatment with 1 mM CPZ, 1 mM CPZ + 15 ng/mL rapamycin or 15 ng/mL rapamycin alone for 24 h and 1 week. **(A,C)** Scale bar: 100 mm. **(B)** Quantification of the mean MBP fluorescence intensity at 24 h and 1 week post-treatment in untreated, CPZ, CPZ + rapamycin or rapamycin alone-treated slices. Data are presented as a percentage of MBP expression in untreated slices 24 h post-treatment. Data represent mean ± SEM with *n* = 3. Statistical significance was determined by a two-way ANOVA with a simple main effect of treatment only and pairwise comparisons performed using Bonferroni’s post-hoc test with ## for *p* < 0.01 and ### for *p* < 0.001 for a significant effect of treatment compared to rapamycin treatment alone. **(D)** Quantification of the mean MOG fluorescence intensity at 24 h and 1 week post-treatment in untreated, CPZ, CPZ + rapamycin or rapamycin alone-treated slices. Data are presented as a percentage of MOG expression in untreated slices 24 h post-treatment. Data represent mean ± SEM with *n* = 3. Statistical significance was determined by a two-way ANOVA with a simple main effect of treatment and pairwise comparisons performed using Bonferroni’s *post-hoc* test with * for *p* < 0.05 and *** for *p* < 0.001 for a significant effect of treatment in comparison to untreated slices and ## for *p* < 0.01 for a significant effect of rapamycin treatment in comparison to CPZ treatment.

To delay CPZ-associated remyelination we treated slices with CPZ in the presence of rapamycin and quantified the expression of MBP and MOG 24 h and 1-week post-treatment. MBP and MOG staining was identified in the corpus callosum in all slices (Figures 4A,C). Quantification of MBP + fluorescence intensity revealed no significant effect of time (*p* = 0.210), but a significant effect of treatment (*p* = 0.0011) on MBP expression. Subsequent *post hoc* analysis revealed a significant effect of CPZ treatment (*p* = 0.009) and CPZ + rapamycin treatment (*p* = 0.0013) when compared to rapamycin treatment alone (Figure 4B). This indicates no further effect of rapamycin on CPZ-induced demyelination.

Quantification of MOG + fluorescence intensity showed no significant effect of time (*p* = 0.424), but a significant effect of treatment (*p* = 0.0005). *Post hoc* analysis revealed a significant effect of CPZ (*p* = 0.0014) and CPZ + rapamycin (*p* = 0.021) treatment in comparison to untreated slices. Additionally, there was a significant difference between CPZ treatment and rapamycin treatment alone (*p* = 0.004) (Figure 4D).

These findings indicate that co-administration of 1 mM CPZ and 15 ng/mL of rapamycin results in the same degree of demyelination seen with 1 mM CPZ treatment alone. Surprisingly, treatment of the slices with rapamycin alone appears to rescue the CPZ-induced demyelination, an opposite effect to what occurs *in vivo*.

### Treatment of rat brain sagittal slice cultures with LPC induces robust demyelination

**Figure 5:**
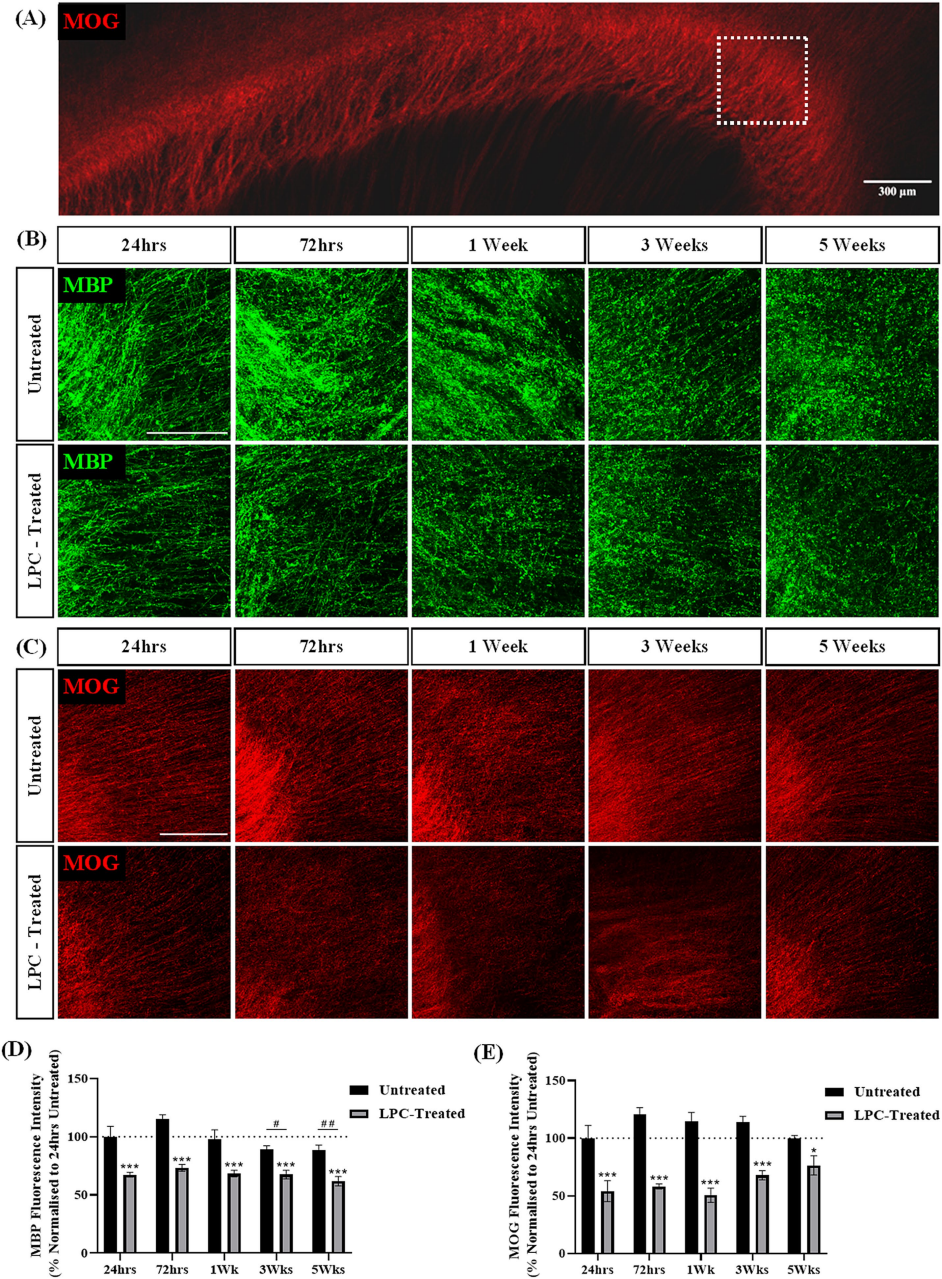
LPC induces demyelination in rat brain sagittal slice cultures up to 5 weeks post-treatment. **(A)** Representative MOG staining of the corpus callosum. The white box indicates the region of interest. Scale bar: 300 mm. **(B)** MBP and **(C)** MOG staining in the corpus callsoum of sagittal rat brain slice cultures following treatment with 0.5 mg/mL LPC or untreated at 24 h, 72 h, 1 week, 3 weeks and 5 weeks post-treatment. Scale bar: 150 mm. **(D)** Quantification of the mean MBP fluorescence intensity up to 5 weeks post-treatment in untreated and LPC-treated slices. Data are presented as a percentage of MBP expression in untreated slices 24 h post-treatment. Data represent mean ± SEM with *n* = 3. Statistical significance was determined by a two-way ANOVA with a simple main effect of treatment and of timepoint with pairwise comparisons performed using Bonferroni’s *post-hoc* test with ***p < 0.001 for a significant effect of LPC treatment in comparison to untreated slices. A significant effect of time irrespective of treatment is depicted as # for *p* < 0.05 and ## for *p* < 0.01 for time point compared to the 72-h time point. **(E)** Quantification of the mean MOG fluorescence intensity up to 5 weeks post-treatment in untreated and LPC-treated slices. Data are presented as a percentage of MOG expression in untreated slices 24 h post-treatment. Data represent mean ± SEM with *n* = 3. Statistical significance was determined by a two-way ANOVA with a significant interaction between time and treatment and pairwise comparisons performed using Bonferroni’s *post-hoc* test with * for *p* < 0.05 and *** for *p* < 0.001.

To assess the effect of a different demyelinating compound on brain slice cultures, slices were treated with LPC for 17 h and the expression of MBP and MOG was assessed over 6 weeks of culture (5 weeks post-treatment). MBP and MOG staining was seen in the corpus callosum of both LPC-treated and untreated slices (Figures 5A–C). LPC-treatment appeared to reduce the intensity of MBP and MOG staining in the corpus callosum at all time points post-treatment (Figures 5B,C). Quantification of MBP + fluorescence intensity revealed a mean MBP fluorescence of 67.38% ± 1.67% at 24 h and 61.83% ± 3.24% at 5 weeks following treatment with LPC compared to 24 h untreated slices. Statistical analysis revealed a significant effect of treatment (*p* = 0.000000003) and a significant effect of time (*p* = 0.009). Subsequent *post hoc* analysis indicated a significant difference in MBP expression at 3 weeks (*p* = 0.034) and 5 weeks (*p* = 0.008) in comparison to 72 h irrespective of treatment (Figure 5D). Quantification of MOG + fluorescence intensity revealed a significant interaction effect of treatment and time (*p* = 0.046). Subsequent post-hoc analysis revealed a significant difference between 24 h untreated and LPC-treated slices at 24 h (LPC: 54.10% ± 7.39%, *p* = 0.0001), 72 h (LPC: 58.24% ± 1.76%, *p* = 0.000002), 1 week (LPC: 50.5% ± 5.03%, *p* = 0.000002), 3 weeks (LPC: 67.95% ± 3.25%, p = 0.0001) and 5 weeks (LPC: 76.43% ± 6.83%, *p* = 0.024) post-treatment (Figure 5E).

Taken together, these results indicate the treatment of sagittal brain slice cultures with 0.5 mg/mL LPC induces demyelination 24 h post-treatment that remains up to 5 weeks post-treatment.

### Treatment of rat brain sagittal slice cultures with LPC induces an increase in GFAP expression that subsides 1 week after treatment

**Figure 6:**
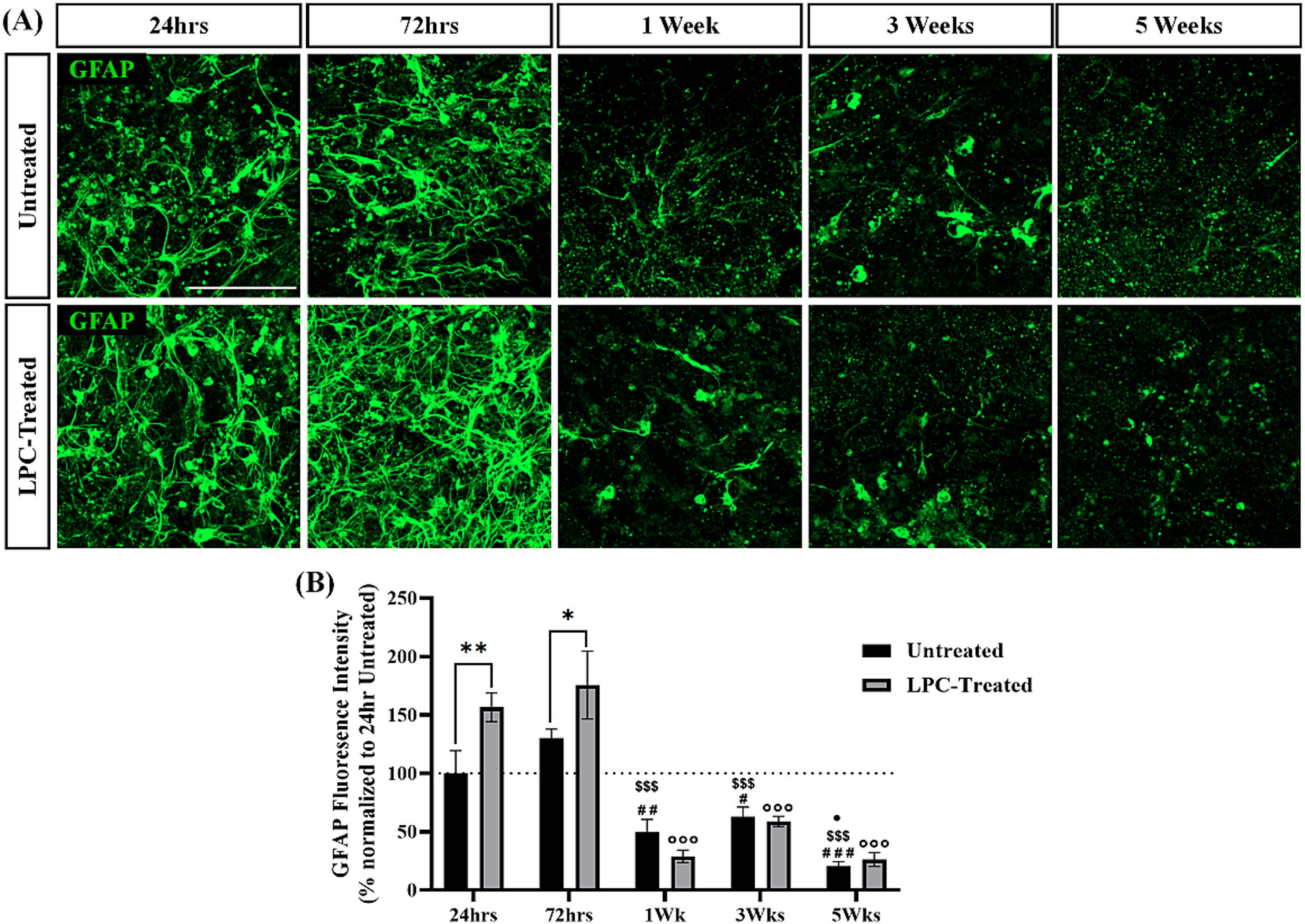
LPC treatment induces an acute increase in GFAP expression that diminishes 1-week post-treatment. **(A)** GFAP staining in the corpus callosum of untreated or LPC-treated sagittal rat brain slice cultures at 24 h, 72 h, 1 week, 3 weeks and 5 weeks post-treatment. Scale bar: 100 mm. **(B)** Quantification of the mean GFAP fluorescence intensity up to 5 weeks post-treatment in untreated and LPC-treated slices. Data are presented as a percentage of GFAP expression in untreated slices 24 h post-treatment. Data represent mean ± SEM with *n* = 3. Statistical significance was determined by a two-way ANOVA with a significant interaction between time and treatment and multiple comparisons performed using Bonferroni’s *post-hoc* test, with * for *p* < 0.05 and ** for *p* < 0.001 for a significant difference of treatment. # for *p* < 0.05, ## for *p* < 0.01 and ### for *p* < 0.001 for a significant difference of untreated slices at a given timepoint compared to 24 h untreated. $$$ for *p* < 0.001 for a significant difference of untreated slices compared to 72 h untreated. **•** For *p* < 0.05 for a significant difference of untreated slices compared to 3 weeks untreated. °°° For *p* < 0.001 for a significant difference of LPC-treated slices compared to 24 h and 72 h LPC-treated.

To further assess the effect of LPC treatment on rat sagittal brain slice cultures, untreated and LPC-treated slices were stained for the astrocyte marker glial fibrillary acidic protein (GFAP) at 24 h, 72 h, 1 week, 3 weeks and 5 weeks post-treatment. GFAP expression was observed in all slices at each timepoint and treatment group (Figure 6A). Quantification of GFAP+ fluorescence intensity revealed a significant interaction of time and treatment (*p* = 0.021, Figure 6B). Subsequent post-hoc analysis demonstrated a significant increase in mean GFAP+ expression following LPC-treatment at 24 h (Untreated: 100% ± 17.6%, LPC-treated: 156.44% ± 11.21%, *p* = 0.0037) and 72 h (Untreated: 129.9% ± 7.12%, LPC-treated: 175.62 ± 26.5%, *p* = 0.017) in comparison to their untreated counterparts. In untreated slices, GFAP expression was reduced at 1 week (49.5% ± 10.02%, *p* = 0.0089), 3 weeks (62.66% ± 7.61%, *p* = 0.049) and 5 weeks (20.43% ± 3.49%, *p* = 0.00008) compared to GFAP expression at 24 h (100% ± 17.6%). Similarly, untreated slices displayed a reduction in GFAP expression at 1 week (*p* = 0.00007), 3 weeks (*p* = 0.0006) and 5 weeks (*p* = 0.0000003) from that at 72 h. A further reduction in GFAP expression was identified from 3 weeks to 5 weeks in untreated slices (*p* = 0.027).

The expression of GFAP in LPC-treated slices displayed a similar trend, with *post hoc* analysis revealing a significant reduction in GFAP expression at 1 week (28.85% ± 4.83%, *p* = 0.000000009), 3 weeks (58.88% ± 4.03%, *p* = 0.000003) and 5 weeks (26.26% ± 5.47%, *p* = 0.000000005) from that at 24 h (156.4% ± 11.2%). Similarly, LPC-treated slices displayed a reduction in GFAP expression at 1 week (*p* = 0.000000000223), 3 weeks (*p* = 0.0000000744) and 5 weeks (*p* = 0.000000000135) from that at 72 h untreated (Figure 6B).

These results demonstrate that GFAP+ expression is high in untreated slice cultures for the first week of culturing and this decreases with prolonged culturing out to 6 weeks (5 weeks post-treatment). Following treatment with 0.5 mg/mL LPC, an initial increase in GFAP+ expression is observed over a 72-h period when compared to that of untreated slices, but with further time in culture, no difference in GFAP+ expression is seen between untreated and LPC-treated slices.

## Discussion

*Ex vivo* organotypic slice culture models of the CNS are valuable for studying IDDs such as MS. In the field, the majority of demyelination studies using slice cultures use cerebellar, hippocampal, or spinal cord tissue, providing useful models of demyelination within these individual regions. However, the use of sagittal organotypic whole brain slices that enable anterior-to-posterior visualization of the corpus callosum is underreported. As the corpus callosum is a region comprised of the heavily myelinated tracts that connect the two hemispheres of the brain, and represents a key site of MS pathology, it is a compelling target for studying demyelination. In this study, we cultured organotypic whole brain slice cultures in the sagittal orientation to visualize the corpus callosum, allowing us to investigate demyelination and remyelination processes. Utilizing this system, we assessed the individual ability of CPZ and LPC to induce demyelination of the corpus callosum and the timing of subsequent remyelination.

CPZ is a commonly used compound to model demyelination in mice, producing reproducible demyelination after 6 weeks of treatment (Hiremath et al., 1998). As such, we investigated the use of CPZ in an *ex vivo* setting to recapitulate the *in vivo* mouse model. The use of CPZ *in vitro* is underreported within the literature, with only a few studies reporting its use. While the primary mechanisms of CPZ toxicity are poorly understood, *in vitro* studies report CPZ toxicity to be limited to primary oligodendrocytes (Bénardais et al., 2013) while others suggest the requirement of pro-inflammatory cytokines (Pasquini et al., 2007). To investigate this, we treated brain slices with CPZ alone, or in the presence of the cytokines TNFa and IFNg, or with the pro-inflammatory compound lipopolysaccharide (LPS), which we have previously demonstrated to induce demyelination in longitudinal organotypic spinal cord slice cultures (Hawker et al., 2024).

We found that treatment of sagittal brain slices with 1 mM CPZ alone induced demyelination of the corpus callosum, reducing MBP expression to ~51% and MOG expression to ~53% of untreated slices, 24 h post-treatment. This contrasts with previous *in vitro* findings that demonstrate the dependence of CPZ toxicity on the presence of exogenous inflammatory activation (Pasquini et al., 2007; Martínez-Pinilla et al., 2021), suggesting that CPZ toxicity *ex vivo* does not require exogenous inflammatory activation. *In vivo* studies link the effects of CPZ not only on demyelination and the death of oligodendrocytes, but also to the concurrent activation of microglia and astrocytes, in turn leading to an elevation in cytokine production (Buschmann et al., 2012; Voß et al., 2012). Hence, it is possible that *in vitro* cell culture studies on CPZ toxicity would require the addition of exogenous cytokines due to the lack of glial cell activation within the culture. However, the enhanced cellular complexity of an *ex vivo* system comes with endogenous production of cytokines, a process demonstrated in previous slice culture experimentation (Dionne et al., 2011; Zou and Crews, 2010) as well as in response to demyelination (O’Sullivan et al., 2016). The endogenous inflammatory activity in *ex vivo* slice cultures may account for the demyelination observed following CPZ treatment in the absence of exogenous pro-inflammatory mediators. However, further studies, including cytokine profiling and analysis of inflammatory mediators are necessary to confirm this hypothesis.

Additionally, it is possible that the concentration and/or duration by which exogenous inflammation was induced in the current study was not efficient in mounting an effective inflammatory response. Interestingly, we found that treating slices with CPZ in combination with cytokines or LPS, appeared to prevent the demyelination seen with CPZ alone. This is an unexpected response and requires further experimentation to understand. The ideal *ex vivo* model of demyelination would provide a platform for testing long-term remyelination-based therapies and hence would need to demonstrate sustained demyelination. As such, we assessed the long-term effect of 1 mM CPZ treatment on myelination of the corpus callosum. We found that at 24 h post-treatment, MBP and MOG expression had dropped to ~48% and ~45%, respectively. However, at 1-week post-treatment, MBP and MOG expression had returned to ~89.3% and ~92%, respectively, indicating a recovery of myelin protein expression, suggestive of remyelination. While no studies have reported the effect of CPZ *ex vivo*, this could be an expected response, based on remyelination seen with CPZ treatment cessation, and is in line with findings from Martinez-Pinilla et al., who demonstrated a recovery in cell viability upon cessation of CPZ treatment *in vitro* (Martínez-Pinilla et al., 2021). Furthermore, the *in vivo* CPZ mouse model suggests a minimum period of 1–2 weeks of CPZ treatment prior to the onset of detectable demyelination (Almuslehi et al., 2020), and hence, perhaps an *ex vivo* response also requires longer treatment exposure for sustained demyelination to be seen. To assess this, we treated slices continuously with 1 mM CPZ for 1 or 2 weeks (‘chronic’ treatment). However, we found no change in MBP or MOG expression in comparison to untreated controls.

As discussed, a key limitation to the use of the CPZ model *in vivo* is the rapid remyelination seen on cessation of treatment (Blakemore, 1972). As such, Sachs et al., generated a model of CPZ in combination with the mTOR inhibitor, rapamycin. The mammalian target of rapamycin (mTOR) pathway is involved in the differentiation of oligodendrocyte precursor cells (OPCs), with inhibition of this pathway through rapamycin treatment shown to block OPC differentiation and decrease expression of myelin proteins (Tyler et al., 2009; Wahl et al., 2014). Through these mechanisms, the combination of CPZ with rapamycin *in vivo* has been shown to delay remyelination, and prolong demyelination, a critical requirement for the testing of remyelination-based therapies (Sachs et al., 2014). To recapitulate this model *ex vivo,* we treated slices with 1 mM CPZ and 15 ng/mL rapamycin continuously for 1 week, in an attempt to sustain CPZ-induced demyelination in the corpus callosum. We found no difference in the expression of MBP or MOG with treatment of slices with CPZ alone or CPZ + rapamycin, suggesting rapamycin had no further effect on the demyelination produced with CPZ treatment alone. Interestingly, there was no difference in MBP or MOG expression over time with treatment of rapamycin only, suggesting rapamycin does not affect myelination *ex vivo*. This was further confirmed by the difference seen in MBP and MOG expression between rapamycin-only and CPZ-only treated slices. This contradicts previous *in vitro* studies on the role of rapamycin on oligodendroglia (Tyler et al., 2009; Ornelas et al., 2020), but again, could be an effect of complex *ex vivo* systems containing multiple cell types, meaning OPCs/OLs may not be a primary target of rapamycin effects *ex vivo* (Tang et al., 2002; Husna et al., 2024). Additionally, a key factor to consider is the timeframe of the current study. As the CPZ + rapamycin *in vivo* model is reported to produce significant myelin loss at 4 weeks of treatment, it is possible that a longer exposure of slices to CPZ + rapamycin could induce the same effect (Sachs et al., 2014).

In this study, we tested multiple treatment paradigms to induce demyelination *ex vivo* using CPZ, however, we were unable to reproduce the sustained demyelination seen *in vivo* (Hiremath et al., 1998). This highlights potential limitations of organotypic slice culture systems, particularly concerning the route of administration and the effective concentration of CPZ. In our *ex vivo* system, CPZ is applied directly to the tissue at a defined concentration, bypassing the physiological processes of absorption and metabolism that occur following oral ingestion *in vivo.* While *in vivo* models use 0.2–0.3% CPZ, the exact blood concentrations are not reported. As a result, the effective concentration required on the cellular level to induce a response is relatively unknown. Further investigation into the metabolic profile of CPZ and active blood concentrations would offer valuable insight into how to induce effective and sustained demyelination in an *ex vivo* setting. Furthermore, as remyelination has been shown to occur in the presence of continuous CPZ exposure *in vivo* (Hiremath et al., 1998), our tested treatment paradigm may likely have been insufficient in either concentration or duration to sustain demyelination and prevent remyelination. Future studies using higher CPZ concentrations and/or extended treatment periods may more effectively model sustained demyelination *ex vivo*. However, as the development and testing of remyelination-based therapies requires effective model systems with sustained demyelination and lack of complete endogenous remyelination, we conclude that 1 mM CPZ treatment of brain slice cultures is not a viable *ex vivo* model for these studies, however, could prove to be useful in acute demyelination studies, pending further characterization.

To build on our previous findings with *ex vivo* longitudinal spinal cord slice cultures (Hawker et al., 2024), we next looked at lysolecithin (LPC) to model sustained demyelination in *ex vivo* brain slice cultures. Treatment of sagittal brain slice cultures with 0.5 mg/mL LPC for 17 h induced robust demyelination of the corpus callosum at 24 h post-treatment, reducing MBP and MOG expression to ~67% and ~54%, respectively. Furthermore, the demyelination seen was sustained with MBP and MOG expression levels of ~61% and ~76%, respectively, at 5 weeks post-treatment. These findings align with previous reports of LPC treatment on cerebellar and spinal cord systems and support the use of LPC as an effective demyelinating compound *ex vivo* (Birgbauer et al., 2004; Hawker et al., 2024). To further characterize the effect of LPC treatment on the corpus callosum in sagittal brain slice cultures, we stained for GFAP to quantify the expression of activated astrocytes. We observed an initial increase in GFAP expression occurring at 24 h and 72 h in all slices, which decreased over time. This response could be a direct effect of slice culture generation. Nevertheless, the GFAP expression at these early timepoints was greater in LPC-treated slices, with an expression of ~156% and ~175% at 24 h and 72 h compared to their untreated counterparts, of 100% and ~130%, respectively. This suggests that the demyelination occurring due to LPC treatment is associated with astrocyte activation, similar to what is proposed to occur *in vivo* (Yoon et al., 2017). Interestingly, 1-week post-treatment, GFAP expression was reduced to ~49% in untreated and ~28.8% in LPC-treated slices, suggesting mitigation of the astroglial response.

In this study, we monitored the expression of myelin proteins MBP and MOG following treatment with CPZ and LPC to assess demyelination. To enable deep tissue imaging, we used the iDISCO tissue clearing method, which renders tissue optically transparent while retaining three-dimensional structure (Renier et al., 2014). However, a key limitation of this protocol is antibody incompatibility. As a result, we were unable to further characterize the temporal dynamics of oligodendrocyte regeneration in response to demyelinating insult. Future studies should address this through staining for markers along the oligodendrocyte lineage, including both immature and mature oligodendrocyte markers, rather than just myelin proteins alone. Additionally, we were unable to assess microglial responses due to the incompatibility of Iba1 antibodies with iDISCO.

Overall, we have demonstrated that rat sagittal whole brain slice cultures are effective tools for modelling acute demyelination and chronic demyelination of the corpus callosum using CPZ or LPC, respectively. We show that treatment of slices with 1 mM CPZ induces acute demyelination that resolves at 1-week post-treatment, but cannot be sustained by chronic administration, nor the addition of rapamycin, as achieved *in vivo*. Conversely, treatment of slices with 0.5 mg/mL LPC for 17 h induces robust demyelination that is sustained up to 5 weeks post-treatment. Furthermore, GFAP expression analysis revealed acute astroglial activation in response to the generation of slice cultures. However, this response is enhanced with LPC treatment and is mitigated by 1 week post-treatment. These findings establish sagittal whole-brain slice cultures as a versatile model for long-term investigation of demyelination and remyelination processes over a 6 weeks in culture. These models provide a valuable *ex vivo* platform for evaluating pharmacological intervention, cell-based therapies and genetic manipulations aimed at enhancing remyelination for the treatment of IDDs.

## Data Availability

The raw data supporting the conclusions of this article will be made available by the authors, without undue reservation.

## Supplementary material

SUPPLEMENTARY FIGURE S1 Sagittal and coronal orientation of slice culture generation. **(A)** Schematic representation of coronal sectioning and **(C)** sagittal sectioning indicating the area of interest, the corpus callosum. MOG staining highlights the corpus callosum, in both **(B)** coronal and **(D)** sagittal slice cultures. Scale bar: 350 μm. ctx: cortex, cc: corpus callosum, str: striatum. Created with https://www.biorender.com/.
