# Supplementary figures and images for: Investigating the use of cuprizone and lysolecithin to model demyelination ex vivo in sagittal rat brain organotypic slice cultures
Source: Front Cell Neurosci. 2025 May 22;19:1609806. doi: 10.3389/fncel.2025.1609806 (PMC12137318; doi:10.3389/fncel.2025.1609806)

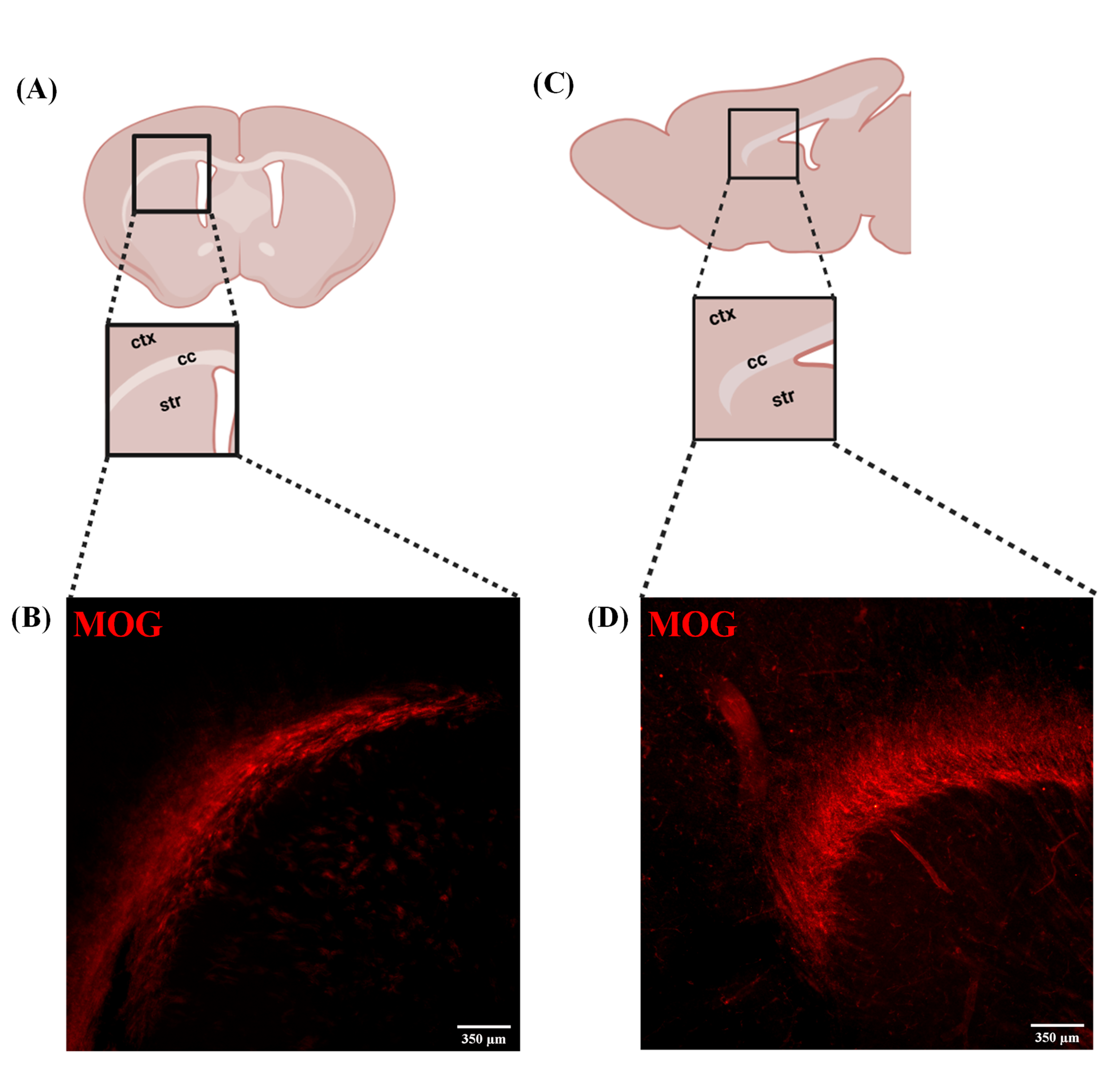

Supplement: SUPPLEMENTARY FIGURE S1 — Sagittal and coronal orientation of slice culture generation. (A) Schematic representation of coronal sectioning and (C) sagittal sectioning indicating the area of interest, the corpus callosum. MOG staining highlights the corpus callosum, in both (B) coronal and (D) sagittal slice cultures. Scale bar: 350 μm. ctx: cortex, cc: corpus callosum, str: striatum. Created with https://www.biorender.com/. [file Image_1.tif]
